# Towards precision agriculture: A dataset for early detection of corn leaf pests

**DOI:** 10.1016/j.dib.2025.111394

**Published:** 2025-02-14

**Authors:** Thierry Tchokogoué, Auguste Vigny Noumsi, Marcellin Atemkeng, Louis Aimé Fono

**Affiliations:** a Laboratory of Computer Science, Faculty of Sciences, University of Douala, Douala BP 24157, Cameroon; b Department of Mathematics, Rhodes University, Artillery Rd, Grahamstown, Eastern Cape 6139, South Africa

**Keywords:** *Spodoptera frugiperda*, *Helminthosporium* leaf blight, *Zonocerus variegatus*, Machine learning, Explainability, Interpretability

## Abstract

Corn (*Zea mays*), commonly referred to as Indian wheat, is a widely cultivated tropical annual herbaceous plant of the Poaceae family. It is primarily grown for its starch-rich grains and as a forage crop. In Cameroon, corn is the most consumed cereal, surpassing rice and sorghum, with an estimated production of 2.2 million tons annually. However, corn production is frequently threatened by insect infestations, which hinder crop development, reduce yields, and degrade its quality. Early detection of insect attacks is essential for farmers, as timely intervention can prevent widespread damage, reduce pesticide usage, and improve production yields. Insect infestations on corn manifest through various symptoms on leaves, stems, and seeds. Among these, foliar attacks are particularly detrimental, disrupting plant growth and significantly reducing yields. Symptoms of these attacks include leaf perforations, yellowing, and white spot deposits, ultimately altering the leaf texture. To address these challenges, machine learning models offer a promising solution for early detection of foliar attacks, enabling farmers to take timely and effective action. This paper introduces a dataset focused on three major pests: Spodoptera frugiperda (Fall Armyworm), *Helminthosporium* leaf blight, and Zonocerus variegatus (Variegated Grasshopper), which are among the most frequent and destructive agents affecting corn crops. The dataset comprises images of corn leaves captured in natural environments at various growth stages and field locations. Images were taken using smartphone cameras at different times of the day, providing diverse lighting conditions, and in various fields, which introduced several background contaminations, ensuring a realistic representation of field conditions. The dataset comprises eight directories: two containing healthy leaf images (1308 without augmentation and 11,772 with augmentation), two containing manually segmented backgrounds of healthy leaves (1308 without augmentation and 11,772 with augmentation), two containing healthy leaves with CNDVI algorithm-segmented backgrounds (1308 without augmentation and 11,772 with augmentation), one containing 848 infected images with manually segmented backgrounds and highlighted infected areas, and one containing 7632 augmented versions of the infected images. This dataset serves as a valuable resource for researchers and students, providing opportunities to develop machine learning and deep learning models for corn disease detection, classification, natural image segmentation, and model interpretability and explainability. By facilitating advancements in precision agriculture and automated pest detection, the dataset contributes to sustainable agricultural practices and the broader field of agroinformatics.

Specifications table

**Table:** 

Subject	Agronomy and Crop Science, Applied Machine Learning
Specific subject area	Application of machine learning in agriculture and crop sciences, insect-attacked corn leaves, precision agriculture and automated pest detection
Type of data	Image
Data collection	Images of corn leaves were captured at different maturation level using mobile devices equipped with high-resolution cameras. The photos were taken in both the morning and evening to capture the leaves under different environmental conditions. Various mobile phones with different camera resolutions were used to capture the images: Samsung Galaxy A22 5G with a 48 MP camera, Tecno Spark 20 with a 50 MP camera, Tecno Camon 20 with a 64 MP camera, Itel W5001P with a 7 MP camera, Tecno F1 with a 7 MP camera, and iPhone 6 with an 8 MP camera.
Data source location	The images were taken under natural lighting conditions with varied backgrounds in crop plantation of several locations in Cameroon: 1. 04 villages of Western Region – Villages of menoua division in west region: • Keleng (Latitude: 5.447922; Longitude: 10.076857) • Dom'meni (Latitude: 5.450000; Longitude: 10.066670) • Baleveng (Latitude: 5.495031; Longitude: 10.153070) • Tsimbing (Latitude: 5.463211; Longitude: 10.082723) 2. Of coastal Region - Wouri division - two: • Logbessou PK17 (Latitude: 4.100316; Longitude: 9.802564) • Papas (Latitude: 4.057849; Longitude: 9.819752) 3. On village of Moungo division in littoral region: Edjocmoa (Latitude: 4.980205; Longitude: 9.946348)
Data accessibility	Repository name: Mendeley Data Data identification number: 10.17632/ymvghfcww7.1 Direct URL to data: https://data.mendeley.com/datasets/ymvghfcww7/1
Related research article	*A robust segmentation method combined with classification algorithms for field-based diagnosis of maize plant phytosanitary state* [1]*.*

## 1 Value of the Data

• The data facilitate early detection and monitoring of major corn leaf diseases. The dataset enables the early identification and continuous monitoring of critical corn leaf diseases such as *Spodoptera frugiperda, Helminthosporium* leaf *blight*, and *Zonocerus variegatus*. By providing high-quality, annotated images of diseased and healthy leaves, researchers and agricultural experts can develop tools to detect diseases at their onset, minimizing crop losses and improving yield management.
• In the context of precision agriculture, this dataset serves as a foundational resource for developing and evaluating advanced image segmentation algorithms and classification models. These tools are essential for automating disease detection, optimizing resource allocation, and enhancing decision-making processes in farming practices.
• Researchers can leverage this dataset to study the explainability and interpretability of machine learning models in agricultural applications. Understanding how models make predictions is crucial for building trust among farmers and stakeholders, ensuring that machine learning and deep learning driven solutions are both effective and transparent.
• The dataset also supports the development of practical monitoring applications for tracking crop health and pest infestations throughout the production cycle. Such tools can provide real-time insights, enabling farmers to take timely actions to mitigate risks and improve crop management.
• The dataset can help develop augmented reality applications for real-time field diagnostics. Farmers can use mobile devices to scan crops, instantly identifying diseases and receiving actionable recommendations. This technology bridges the gap between advanced research and on-ground agricultural practices, making disease detection more accessible and efficient.
• The dataset is invaluable for studying plant phenotyping, which involves analyzing plant traits and growth patterns. It also facilitates the development of automated systems using machine learning and deep learning for monitoring crop growth stages, enabling researchers to track plant development, optimize breeding programs, and improve crop resilience.

## 2 Background

Corn's growing importance across food production, animal feed, and human consumption makes protecting crops from pest infestations crucial [2]. Insect attacks on corn significantly affect yields, manifesting through visible changes in leaf texture and color. Early detection of these symptoms provides critical opportunities for intervention and supports sustainable agriculture practices [[3], [4], [5], [6]].

Early field diagnosis of corn plant health faces challenges due to variable environmental backgrounds that can affect classification algorithm performance. While the work in [1] focused on background segmentation algorithms for corn plants, this paper extends existing datasets to include specimens affected by Spodoptera frugiperda, *Helminthosporium* leaf blight, and Zonocerus variegatus. These specific diseases were selected based on their documented economic impact on corn production and their distinct visual signatures on leaves, making them suitable candidates for machine learning models.

**Fig. 1:**
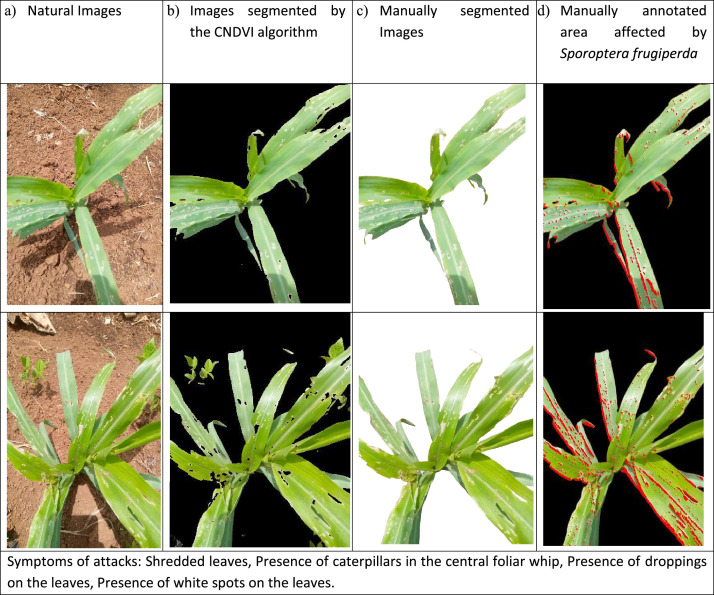
Examples of images of corn leaves that have undergone an attack by *Spodoptera frugiperda.*

*Spodoptera frugiperda* [7,8] is commonly known as the fall armyworm, is a highly destructive pest that poses a serious threat to numerous crops, especially corn. This invasive species can lead to significant crop losses, impacting food security and causing economic damage in agricultural regions worldwide. Adult moths lay eggs on leaves, and upon hatching, larvae begin feeding on leaf tissue, creating characteristic transparent windows and ragged holes. As larvae develop through six instars over 14–21 days, they can completely defoliate plants and destroy growing points, leading to yield losses of 20–50 % in severe cases. Current management practices include chemical pesticides, biological control agents, and resistant varieties, but the pest's rapid reproduction rate and growing pesticide resistance pose significant challenges. Early detection is crucial as control measures are most effective in early larval stages, making this dataset valuable for developing automated monitoring systems. Fig. 1a shows corn images affected by *Spodoptera frugiperda* taken in a natural environment, Fig. 1b–d show the same corn image where the background of the images are segmented by the CNDVI algorithm, background of the images are manually segmented and where the area affected by *Spodoptera frugiperda* is annotated, respectively.

**Fig. 2:**
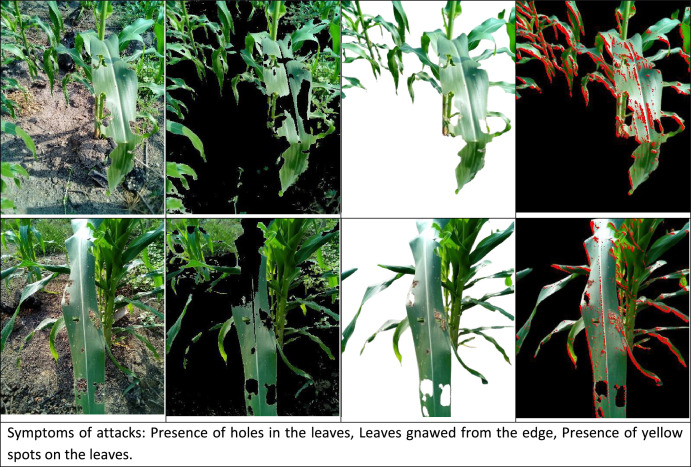
Examples of images of corn leaves that have been attacked by *Zonocerus Variegatus*.

*Zonocerus variegatus* [9,10] is commonly known as the variegated grasshopper, is a significant agricultural pest notorious for its destructive feeding habits on crops, particularly corn. The variegated grasshopper's feeding activity can cause extensive defoliation, compromising the plants' ability to carry out photosynthesis, which, in turn, affects growth and significantly reduces crop yields. Under favorable conditions (moderate temperatures and high humidity), the disease can spread rapidly through fields via wind-dispersed spores, leading to extensive leaf damage, reduced photosynthetic capacity, and yield losses of up to 40 %. Current control strategies rely heavily on fungicide applications, crop rotation, and resistant hybrids. However, the disease's ability to overcome host resistance and the lack of early warning systems highlight the need for improved detection methods, which this dataset aims to address through machine learning applications. Fig. 2a shows corn images affected by *Zonocerus variegatus* taken in a natural environment, Fig. 2b–d show the same corn image where the background of the images are segmented by the CNDVI algorithm, background of the images are manually segmented and where the area affected by *Zonocerus variegatus* is annotated, respectively.

**Fig. 3:**
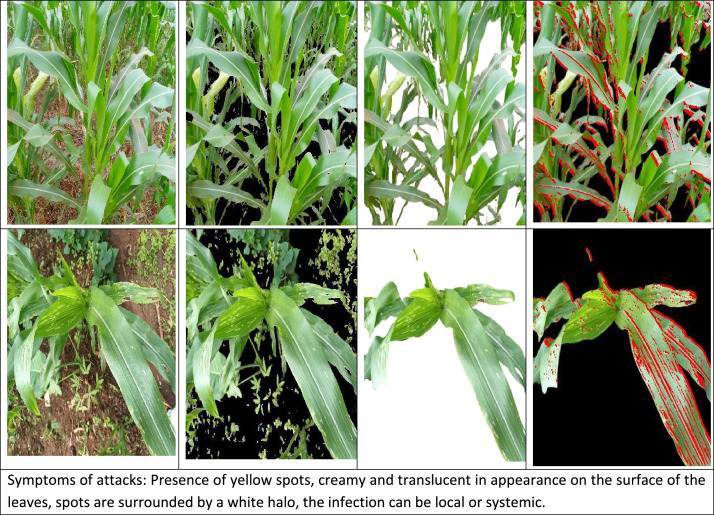
Examples of images of corn leaves that have suffered an attack of *Helminthosporiosis*.

*Helminthosporiose* [11] is a fungal disease that affects a variety of crops, including corn. It is caused by fungi of the genus *Helminthosporium*, which can cause leaf spotting and scorching on affected plants. Symptoms on corn leaves typically include small, tan-colored elliptical spots that can expand and coalesce, causing significant damage to the foliage. This disease can have a significant impact on crop yield and quality. Traditional control methods include chemical insecticides, biological control, and cultural practices such as destruction of egg-laying sites. However, the grasshopper's broad host range and increasing resistance to conventional insecticides necessitate improved monitoring and early intervention strategies. The inclusion of this pest in the dataset provides essential visual data for developing automated detection systems that can alert farmers to early-stage infestations when control measures are most effective. Fig. 3a shows corn images affected by *Helminthosporiose* taken in a natural environment, Fig. 3b–d show the same corn image where the background of the images are segmented by the CNDVI algorithm, background of the images are manually segmented and where the area affected by *Helminthosporiose* is annotated, respectively.

**Fig. 4:**
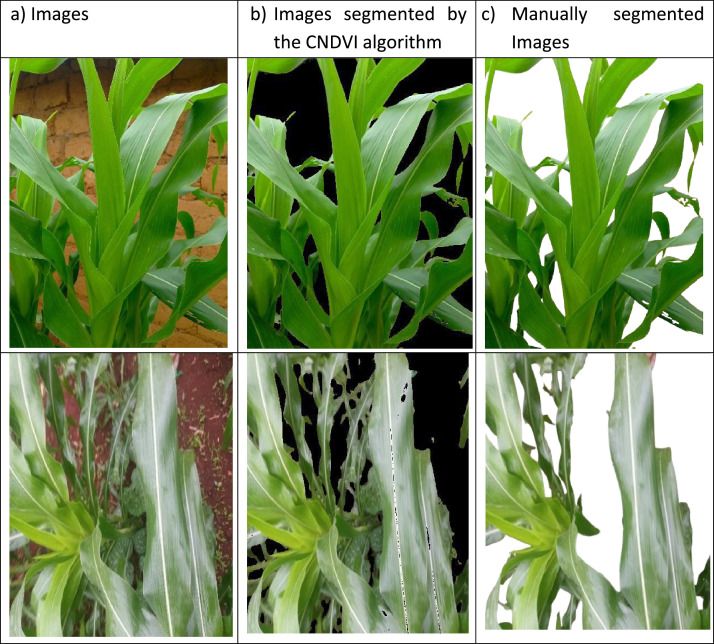
Examples of images of healthy corn leaves.

To sum up, Fig. 1, Fig. 2, Fig. 3 show examples of images of corn leaves that have undergone attack by each pest, whereas Fig. 4 presents examples of images of healthy corn leaves.

## 3 Data Description

**Fig. 5:**
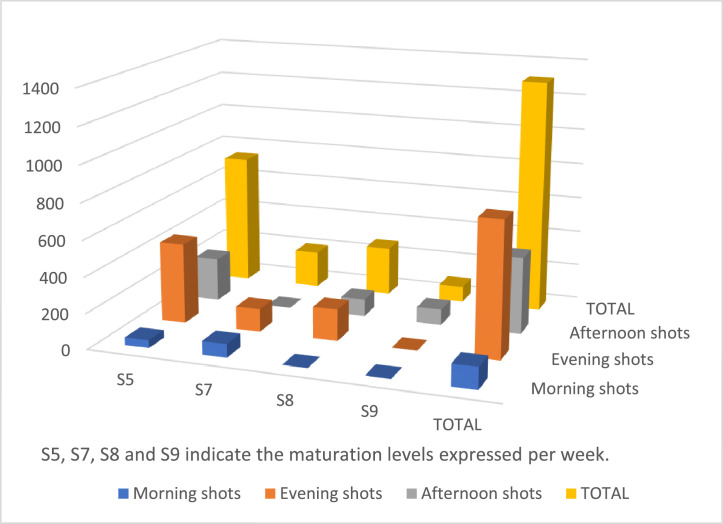
Maturation level versus the distribution of images collected during morning, afternoon, and evening.

The dataset was collected from several locations in Cameroon and consists of images of healthy corn leaves photographed against various backgrounds and at different maturation states during various times of the day. Table 1 and Fig. 5 provide quantitative measures of the maturation levels and the distribution of the corn images collected during the morning, afternoon, and evening. Our data collection methodology followed established plant pathology principles and agricultural monitoring practices. We implemented standardized protocols for documenting plant diseases, ensuring images captured key diagnostic features used by experts in traditional field assessments. The sampling strategy incorporated various corn maturation states and different times of the day under diverse field conditions to ensure a comprehensive representation of disease manifestations.

**Table 1:** Distribution of images collected during morning, afternoon and evening with their maturation level.

Maturation	Morning shots	Evening shots	Afternoon shots	Total
S5	44	451	243	738
S7	77	129	0	206
S8	0	177	98	275
S9	0	0	89	89
TOTAL	121	757	430	1308

The first phase of this work involved acquiring corn plant images. As suggested in [12], images could be of the entire plant, a leaf, flower, stem, or fruit. They identified three categories of images based on acquisition method: ``Scan,'' ``Pseudo-scan,'' and ``Photos.'' In scan and pseudo-scan categories, leaf images are captured through scanning and photography processes respectively, meaning images are taken against a uniform colored background under fixed lighting conditions. For the third category, images are of plants captured in their natural environment (with varying backgrounds and lighting conditions). For this phase, we manually visited several fields in two regions of Cameroon (see details in Table 4) and collected 1,308 images of corn leaves in their natural environment at various times of day (morning, afternoon, and evening) and at several maturity stages (Table 1 and Fig. 5) using smartphone cameras. The codes S5 to S9 indicate corn plant maturity stages, with S5 representing images taken five weeks after sowing, S6 after six weeks, etc. Including multiple maturity stages allows machine learning and deep learning models to learn stage-specific features and improves their ability to recognize plants at different growth phases. The entire image dataset was annotated by two agricultural experts from MINADER (Ministry of Agriculture and Rural Development of Cameroon). These experts organized the images into four classes:

- Corn images containing only healthy leaves
- Corn images containing leaves infected by *Spodoptera frugiperda* (M1)
- Corn images containing leaves infected by *Helminthosporium leaf blight* (M2)
- Corn images containing leaves infected by *Zonocerus variegatus* (M3)

To facilitate the identification or location of each image in the image dataset, an encoding format was defined and applied to all images in the dataset. Table 2 provides a description of the different encoding format attributes. For example, the code C1M1S5-5_03_24-1 assigned to an image indicates corn leaf taken in field 1 (C1), attacked by *Spodoptera frugiperda* (M1), in its fifth week of growth (S5), on March 5, 2024 (05_03_24), and numbered as 1.

**Table 2:** Description of each element contained in the image encoding format.

State	Indicates the condition of the leaves	*Healthy (S), Spodoptera frugiperda (M1), Helminthosporium leaf blight (M2), Zonocerus variegatus (M3)*
Maturity	Indicates the age of the plant	S*i; i=1,…, N*
Field	Indicates the field were the image was collected	C1 (Field 1), C2(Field 2), …, C*i* (Field *i*)
Picture Date	Indicates the date and month when the image was captured	Month_Day_Year. Example 10_05_24 (May, 10, 2024)
Number	Indicates the number of the image	1, 2, 3, …, M

Additionally, the dataset includes images of corn leaves affected by *Spodoptera frugiperda, Helminthosporium* leaf blight, and *Zonocerus variegatus*. Table 3 provides descriptions of the symptoms of infections caused by *Spodoptera frugiperda, Zonocerus variegatus*, and *Helminthosporiosis*, while Table 4 shows the distribution of samples across classes, collection locations, and sample quantities.

**Table 3:** Decease attack and symptoms.

Sr. No	Species Name	Symptoms
01	*Sporoptera frugiperda* [7,8]	- Shredded leaves, - Presence of caterpillars in the central leaf rosette, - Presence of droppings on the leaves, - Presence of white spots on the leaves.
02	*Zonocerus Variegatus* [9,10]	- Presence of holes in the leaves - Leaves gnawed from the edge - Presence of yellow spots on the leaves
03	*Helminthosporiose* [11]	- Presence of yellow spots, creamy and translucent in appearance, on the surface of the leaves. These spots are surrounded by a white halo. - The infection can be either localized or systemic.

**Table 4:** Distribution of samples across classes, collection locations, and sample quantities (La and Lo refer to latitude and longitude respectively).

Sr. No	Location	Species Name	Infected	Healthy
1	Keleng (Menoua division; La: 5.447922; Lo: 10.076857)	*Sporoptera frugiperda*	243	0
2	Dom'meni (Menoua division; La: 5.450000; Lo: 10.066670)	*Sporoptera frugiperda*	249	246
3	Baleveng (Menoua division; La: 5.495031; Lo: 10.153070)	*Helminthosporiose*	114	15
4	Tsimbing1 (Menoua division; La: 5.463211; Lo: 10.082723)	*Sporoptera frugiperda*	75	34
5	Tsimbing2 (Menoua division; La: 5.463211; Lo: 10.082723)	*Sporoptera frugiperda*	20	78
6	Logbessou Papas1 (Wouri division; La: 4.057849; Lo: 9.819752)	*Zonocerus Variegatus*	45	44
7	Logbessou Papas2 (Wouri division; La: 4.057849; Lo: 9.819752)	*Sporoptera frugiperda*	26	14
8	Logbessou PK17 (Wouri division; La: 4.100316; Lo: 9.802564)	*Sporoptera frugiperda*	45	23
9	Edjocmoa (Moungo division; La: 4.980205; Lo: 9.946348)	*Helminthosporiose*	31	6
10	Totals		848	460
11	Total		1308	

The raw captured images were resized, and unwanted backgrounds were automatically masked. To enhance the richness of the datasets, each subset of the dataset was augmented by a factor of 9, with the potential for further augmentation to improve model training. The dataset consists of eight labeled directories described as follows:

• **Directory 1** contains 1308 natural images captured in different fields and all resized to 400 × 400 pixels. In addition, **Directory 4** contains 11,772 images augmented version and corresponding to a 9-fold increase in the content of Directory 1
• **Directory 2** contains 1308 images with manually segmented backgrounds i.e. the background is masked in black. In addition, **Directory 5** contains 11,772 manually segmented images augmented version and corresponding to a 9-fold increase in the content of Directory 2.
• **Directory 3** contains 1308 images with automatically segmented backgrounds using the new CNDVI-based algorithm (see [1]), i.e., the background is masked. In addition, **Directory 6** contains 11,772 automatically segmented images augmented version and corresponding to a 9-fold increase in the content of Directory 3.
• **Directory 7** contains 848 images of infected plants, with manually segmented backgrounds and highlighted infected areas. In addition, **Directory 8** contains 7632 images augmented version and corresponding to a 9-fold increase in the content of Directory 7.

## 4 Experimental Design, Materials and Methods

To create a comprehensive dataset of corn leaf attacks, images were captured using various mobile cameras. Table 5 details the camera specifications and image quality. These devices offer a range of camera resolutions, ensuring a varied dataset that can be useful for different machine learning applications. Images were taken under a variety of environmental conditions, with most captured in the morning and evening to account for different lighting and shading effects. Some images were also taken in the afternoon, which introduces natural shading and allowed for a broader representation of visual conditions. In the early morning images, dew is often present on the corn leaves, adding additional texture and complexity to the dataset.

**Table 5:** Camera specifications.

N°	Camera Specifications	Samsung Galaxy A22 5G	Tecno Spark 20	Tecno camon 20	Itel W5001P	Tecno F1	IPhone6
1	Mega Pixel	48	50	64	7	7	8
2	Dimension	6000 × 8000	6129 × 8160	4032 × 8960	1920 × 2560	1920 × 2560	2448 × 3264
3	Horizontal Resolution	6000	8160	4032	1920	1920	2448
4	Vertical Resolution	8000	1612	8960	2560	2560	3264
5	Bit Depth	96	96	96	96	96	96

The images were gathered from diverse villages across Cameroon, representing different soil types, climates, and agricultural practices. This diversity ensures that the dataset is robust and can be applied to various real-world applications. With the aid of agricultural experts and farmers in the field, the dataset was labeled to ensure accuracy and reliability. Labeling involved identifying healthy and diseased corn leaves, as well as categorizing the specific types of diseases. The process included thorough visual inspection and expert annotation to highlight critical features of each disease. This collaborative approach ensured that the dataset not only represented real-world conditions but also minimized errors in categorization. However, challenges such as variability in disease presentation across different environmental conditions and corn maturity stages required careful deliberation and multiple rounds of validation. The involvement of domain experts was particularly critical in distinguishing subtle differences between similar symptoms caused by different pests, thereby enhancing the dataset's robustness for machine learning applications.

The dataset underwent several preprocessing and augmentation stages to create a comprehensive collection suitable for various machine learning applications. Initially, all 1308 natural images were standardized to 400 × 400 pixels using MATLAB's imresize() function (Directory 1), ensuring consistency in image dimensions for subsequent processing steps. The segmentation process was conducted through two parallel approaches. The first approach involved manual refinement using the online tool remove.bg [13] and Adobe Photoshop version 23.5.5 (2023). This process eliminated all foreign elements from each image, isolating corn leaves against a uniform background (Directory 2). The second approach implemented an automated segmentation technique based on CNDVI [1]. This novel method automatically removed backgrounds from each image, isolating corn foliage against a black background (Directory 3), demonstrating the potential for automated preprocessing in large-scale agricultural image analysis.

For images containing infected leaves, an additional segmentation layer was applied using edge detection techniques with Sobel filters [citation]. This process highlighted infected regions in red, precisely highlighting areas affected by pests (Directory 7). This specialized segmentation provides crucial information for training models to identify specific disease patterns and infection characteristics.

To enhance model robustness and enable deep learning applications, each directory underwent extensive data augmentation using various techniques including rotation, zooming, and resizing. This process significantly expanded the dataset:

- Directory 4: 11,772 augmented natural images
- Directory 5: 11,772 images with manual segmentation
- Directory 6: 11,772 images with automated CNDVI segmentation
- Directory 8: 7,632 images with highlighted infected areas

**Fig. 6:**
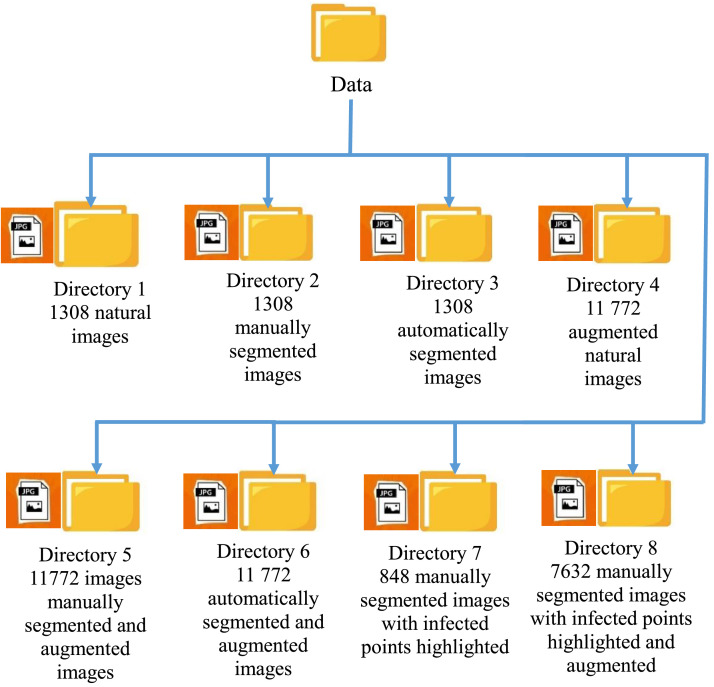
Dataset structure.

The hierarchical structure of the dataset (Fig. 6) and as uploaded in [14] ensures systematic organization of both original and augmented images across different preprocessing stages. This comprehensive organization facilitates various research applications, from comparing segmentation techniques to developing automated disease detection systems. The multiple preprocessing approaches and augmentation strategies provide researchers with flexibility in choosing appropriate data representations for their specific machine learning and deep learning tasks, whether focusing on raw image analysis, segmented leaf studies, or infection pattern recognition. The combination of manual and automated segmentation methods provides valuable benchmarks for developing improved automated preprocessing pipelines for agricultural imaging applications.

The datasets in [15,16] featuring corn pest diseases like Maize Lethal Necrosis and Maize Streak Virus, and in [17] focusing on diseases like Northern Leaf Blight, or [18] which includes Fall Armyworm, Grasshopper, Leaf Beetle, Leaf Blight, Leaf Spot, and Streak Virus. These existing datasets lack certain critical features. For instance, they do not highlight infected areas of the corn leaves, do not provide manual background segmentation, and do not include a baseline published algorithm for background segmentation that researchers can use for comparison. The absence of detailed annotations for infected regions limits the ability to train and evaluate advanced machine learning and deep learning models, particularly those relying on precise localization of disease symptoms. Manual background segmentation, which is crucial for isolating the leaf from complex backgrounds, is also missing in these datasets. This omission hinders the development of robust segmentation models that can operate effectively in real-world agricultural settings, where leaves are often surrounded by soil, stems, or other plants. Additionally, the lack of a baseline algorithm for background segmentation makes it challenging for researchers to benchmark their work against a standardized method. Our dataset includes a diverse range of disease stages and environmental conditions, ensuring that models trained on it are robust and capable of handling real-world variability. This is particularly important for applications in precision agriculture, where early and accurate detection of diseases can significantly impact crop yield and food security.

## Limitations

Compared [[15], [16], [17]], our dataset currently offers a limited number of samples. Collecting more image samples from other regions of Cameroon and even abroad would enhance the quality and diversity of the dataset, as well as increase its overall size.

## Ethics Statement

The authors confirm that this work does not involve human subjects, animal experiments, or any data collected from social media platforms.

## CRediT authorship contribution statement

**Thierry Tchokogoué:** Conceptualization, Methodology, Investigation, Data curation, Validation, Writing – original draft, Visualization. **Auguste Vigny Noumsi:** Conceptualization, Validation, Resources, Writing – review & editing, Supervision. **Marcellin Atemkeng:** Conceptualization, Methodology, Validation, Resources, Writing – original draft, Writing – review & editing, Visualization, Funding acquisition. **Louis Aimé Fono:** Conceptualization, Methodology, Validation, Resources, Writing – review & editing, Supervision, Visualization, Funding acquisition.

## Data Availability

Mendeley DataA Dataset for Early Detection of Corn Leaf Pests in Precision Agriculture (Original data).

## References

[1] Tchokogoué T., Noumsi A.V., Atemkeng M., Fonkou M.F.Y., Fono L.A. (2024). A robust segmentation method combined with classification algorithms for field-based diagnosis of maize plant phytosanitary state. J. Intell. Syst..

[2] Ranum P., Peña-Rosas J.P., Garcia-Casal M.N. (2014). Global maize production, utilization, and consumption. Ann. N. Y. Acad. Sci..

[3] Gilligan C.A. (2008). Sustainable agriculture and plant diseases: an epidemiological perspective. Philos. Trans. R. Soc. B Biol. Sci..

[4] Suryawanshi Y., Patil K., Chumchu P. (2022). VegNet: dataset of vegetable quality images for machine learning applications. Data Br..

[5] Patil K., Suryawanshi Y., Patrawala A., Chumchu P. (2024). A comprehensive lemongrass (*Cymbopogon citratus*) leaf dataset for agricultural research and disease prevention. Data Br..

[6] Thite S., Patil K., Jadhav R., Suryawanshi Y., Chumchu P. (2024). Empowering agricultural research: a comprehensive custard apple (Annona squamosa) disease dataset for precise detection. Data Br..

[7] Andrews K.L. (1980). The whorlworm, *Spodoptera frugiperda* in Central America and neighboring areas. Fla. Entomol..

[8] Paredes-Sánchez F.A., Rivera G., Bocanegra-García V., Martínez-Padrón H.Y., Berrones-Morales M., Niño-García N., Herrera-Mayorga V. (2021). Advances in control strategies against *Spodoptera frugiperda*. A review. Molecules.

[9] Modder W.W.D. (1994). Control of the variegated grasshopper Zonocerus variegatus (L.) on cassava. Afr. Crop Sci. J..

[10] Thomas M.B., Jenkins N.E. (1997). Effects of temperature on growth of Metarhizium flavoviride and virulence to the variegated grasshopper, Zonocerus variegatus. Mycol. Res..

[11] Nisikado Y., Miyake C. (1921).

[12] Wäldchen J., Mäder P. (2018). Using deep learning for image-based plant disease detection. Arch. Comput. Methods Eng..

[13] Sunitha P., Uma B., Channakeshava S, Suresh Babu; (2023). A fully labelled image dataset of banana leaves deficient in nutrients. Data Br..

[14] Tchokogoué, Thierry; Noumsi, Auguste; Atemkeng Teufack, Marcellin; FONO, LOUIS AIME (2024), “A Dataset for Early Detection of Corn Leaf Pests in Precision Agriculture”, Mendeley Data, V1. 10.1016/j.dib.2025.111394 PMC11905841 40083639

[15] Mduma N., Laizer H., Loyani L., Macheli M., Msengi Z., Karama A., Msaki I., Sanga S. (2022). Maize dataset Tanzania. Havard dataverse. Data Br..

[16] Mduma N., Mayo F. (2024). Updating “machine learning imagery dataset for maize crop: a case of Tanzania” with expanded data to cover the new farming season. Data Br..

[17] Wiesner-Hanks T., Stewart E.L., Kaczmar N. (2018). Image set for deep learning: field images of maize annotated with disease symptoms. BMC Res. Notes.

[18] Mensah P.K., Akoto-Adjepong V., Kwabena A., Ayidzoe M.A., Elvis Asare Bediako E.A., Nyarko-Boateng O., Boateng S., Esther Fobi Donkor E.F., Bawah F.U., Nicodemus Songose Awarayi N.S., Nimbe P., Nti I.K., Abdulai M., Adjei R.R., Opoku M., Abdulai S., Amu-Mensah F. (2023). CCMT: dataset for crop pest and disease detection. Data Br..

